# Selective Fetoscopic Laser Photocoagulation of Placental Anastomoses Leads to Early Reduction of Cardiovascular Burden in the Twin‐Twin Transfusion Syndrome

**DOI:** 10.1002/pd.6805

**Published:** 2025-04-26

**Authors:** Kasey J. Chaszczewski, Sarah Perelman, Christine B. Falkensammer, Anita Szwast, Chengcheng Pang, Zhiyun Tian, Juliana Gebb, Julie Moldenhauer, Nahla Khalek, Jack Rychik

**Affiliations:** ^1^ Herma Heart Institute Children's Wisconsin Milwaukee Wisconsin USA; ^2^ Department of Pediatrics Medical College of Wisconsin Milwaukee Wisconsin USA; ^3^ Perelman School of Medicine at the University of Pennsylvania Philadelphia Pennsylvania USA; ^4^ Fetal Heart Program at the Cardiac Center at The Children's Hospital of Philadelphia Philadelphia Pennsylvania USA; ^5^ The Richard D. Wood Jr. Center for Fetal Diagnosis and Treatment The Children's Hospital of Philadelphia Philadelphia Pennsylvania USA; ^6^ Guangdong General Hospital and Guangdong Cardiovascular Institute Guangzhou China

**Keywords:** cardiomyopathy, fetal echocardiography, monochorionic twins, selective fetoscopic laser photocoagulation, twin‐twin transfusion syndrome

## Abstract

**Objective:**

Cardiovascular disturbances are common in twin‐twin transfusion syndrome (TTTS). However, the rate of improvement in cardiovascular burden in response to selective fetoscopic laser photocoagulation (SFLP) is not well known.

**Method:**

Fetal echocardiograms were performed prior to and 1 week following SFLP. Cardiovascular burden was characterized using the Children's Hospital of Philadelphia (CHOP) TTTS Cardiovascular (CV) Score. Pulsatility indices (PI) of the umbilical artery (UA) and middle cerebral artery (MCA), cerebroplacental ratio (CPR) and elements of CHOP CV Score were analyzed pre and post SFLP.

**Results:**

SFLP was performed in 198 subjects; 17 were excluded due demise post SFLP. Following SFLP, recipient (R) demonstrated an increase in MCA PI and a decrease in UA PI, with an increase in CPR. Donor (D) demonstrated a similar magnitude decrease in MCA PI and UA PI, yielding no change in CPR. Following SFLP, the mean CHOP CV Score decreased. The magnitude of change was greater in the subgroup with greater pre‐SFLP cardiovascular burden (CHOP CV Score ≥ 6).

**Conclusion:**

Improvement in CV burden is seen as early as 1‐week post‐SFLP, supporting acute alteration of loading conditions as a significant contributor. Further study of the trajectory of CV alterations may provide insight into the complex mechanisms underlying TTTS.

**Summary**

- What's already known about this topic?
  ◦ In monochorionic pregnancies impacted by twin‐twin transfusion syndrome (TTTS), there is limited knowledge of the acute impact of selective fetoscopic laser photocoagulation (SFLP) on cardiovascular characteristics.
  ◦ Understanding the transition of the fetal cardiovascular state in response to SFLP may offer insight into the pathophysiology of TTTS.
- What does this study add?
  ◦ Following SFLP, recipient fetuses demonstrated an increase in middle cerebral artery (MCA) pulsatility index (PI) and a decrease in umbilical artery (UA) PI. Donor fetuses demonstrated a similar magnitude decrease in MCA PI and UA PI.
  ◦ Changes in the CHOP TTTS Cardiovascular Score following SFLP support the flow related phenomena of TTTS with the most common elements to improve being systolic dysfunction, tricuspid inflow, ductus venosus and umbilical vein flow patterns.

## 1 Introduction

Twin‐twin transfusion syndrome (TTTS) develops in 10%–15% of monochorionic pregnancies and is a major contributor to morbidity and mortality in twins [1]. Intrinsic to the pathophysiology of TTTS are placental anastomoses that lead to maldistribution of blood volume and a unique cascade of events resulting in alterations of cardiovascular loading conditions for each fetus [2]. The Quintero staging system is designed as a clinical scoring system to categorize TTTS severity [3]. However, exclusive utilization of Quintero staging fails to sufficiently account for the specific type and magnitude of cardiovascular abnormalities experienced in this condition. For this reason, as a complement to the Quintero staging system, the Children's Hospital of Philadelphia (CHOP) TTTS Cardiovascular (CV) Score was developed to specifically assess cardiovascular burden in fetuses with TTTS [4].

While there is discussion surrounding the optimal management strategy for pregnancies with Quintero Stage I TTTS, selective fetoscopic laser photocoagulation (SFLP) of intertwin anastomoses is first line therapy in pregnancies impacted by TTTS Stage II or greater [5]. SFLP therapy improves survival, as well as postnatal neurodevelopmental outcomes [5, 6]. Cardiovascular abnormalities are inherently part of the disease presentation in TTTS with residual manifestations in survivors of SFLP therapy noted in childhood and potentially lifelong [7, 8, 9, 10]. Although SFLP therapy inherently alters physiology, studies of the acute impact on specific cardiovascular characteristics are limited [11]. Exploring the acute transition of the fetal cardiovascular state in response to SFLP may offer novel insights into the pathophysiology of TTTS and recovery following treatment. In this study, we characterize the early impact of SFLP on cardiovascular burden in TTTS through serial assessment of vascular indices and application of the CHOP TTTS CV Score [4] with analysis of individual elements.

## 2 Methods

The study was approved by the Institutional Review Board of The Children's Hospital of Philadelphia and a waiver of consent was granted. A single‐center retrospective review was performed in a consecutive series of patients referred with a diagnosis of TTTS to The Richard D. Wood Jr. Center for Fetal Diagnosis and Treatment at CHOP. The study protocol was reviewed and approved by the Children's Hospital of Philadelphia Institutional Review Board (IRB 11‐008076). SFLP was performed in a standard fashion, ablating all donor‐recipient anastomoses on the surface of the placenta [12]. SFLP was offered in cases of Quintero Stage II‐IV and in select cases with Stage I TTTS at an increased risk of progression or pregnancy loss (i.e. severe polyhydramnios or CHOP TTTS CV Score > 5).

Fetal echocardiograms performed prior to and 1 week post SFLP were reviewed. Extracted clinical data elements included: umbilical artery (UA) pulsatility index (PI), middle cerebral artery (MCA) PI and cerebroplacental ratio (CPR) for both twins. The total CHOP TTTS CV Score, as well as individual contributory elements, were recorded (Table 1). The CHOP TTTS CV Score is a 20‐point cardiovascular assessment that encompasses a total of five domains, four in the recipient and one in the donor. Variables in the recipient fetus include abnormalities of: (1) ventricular hypertrophy and systolic dysfunction, (2) atrioventricular valve insufficiency, (3) diastolic performance through ventricular inflow, ductus venosus and umbilical vein flow patterns, (4) right ventricular outflow/pulmonary valve obstruction; and in the donor fetus abnormalities of (5) umbilical arterial flow. Score elements are either semiquantitative or qualitative with definitions as previously described [4]. Scores were assigned at the time of fetal echocardiogram by a small group of experienced, dedicated specialists (JR, ZT) trained to create consistency and highly familiar with the grading system. Data were collected from the original reports. Subjects without complete echocardiographic data were excluded as were subjects with demise of either fetus to permit paired analyses of donor and recipient survivors.

**TABLE 1:** Elements of the CHOP CV score.

	Parameter	Finding	Numeric score
Recipient	Ventricular hypertrophy	None	0
		Present	1
	Cardiac dilation	None	0
		Mild	1
		> Mild	2
	Ventricular dysfunction	None	0
		Mild	1
		> Mild	2
	Tricuspid valve regurgitation	None	0
		Mild	1
		> Mild	2
	Mitral valve regurgitation	None	0
		Mild	1
		> Mild	2
	Tricuspid valve inflow	Double‐peak	0
		Single‐ peak	1
	Mitral valve inflow	Double‐peak	0
		Single‐peak	1
	Ductus venosus	All antegrade	0
		Absent diastolic blood flow	1
		Reverse diastolic blood flow	2
	Umbilical vein	No pulsations	0
		Pulsations	1
	Right sided outflow tract	Pulmonary artery > aorta	0
		Pulmonary artery = aorta	1
		Pulmonary artery < aorta	2
		Right ventricular outflow tract obstruction	3
	Pulmonary regurgitation	None	0
		Present	1
Donor	Umbilical artery	Normal diastolic blood flow	0
		Decreased diastolic blood flow	1
		Absent/reversed diastolic blood flow	2

Mean and 95% confidence intervals were calculated for UA PI, MCA PI, CPR and CHOP TTTS CV Score elements. Values were analyzed pre and post SFLP using paired t‐tests. Subgroup analyses of subjects with a CHOP TTTS CV Score ≥ 6 was undertaken to assess the impact of SFLP in a cohort with a greater magnitude of cardiovascular abnormality at presentation to explore the response to SFLP. *p* value < 0.05 was considered of significance.

## 3 Results

**FIGURE 1:**
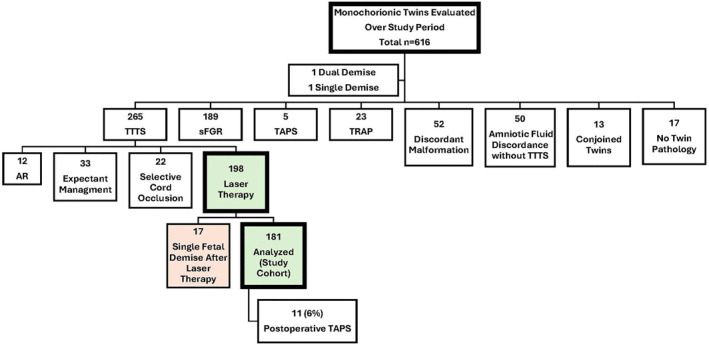
Monochorionic twin pregnancies during study period: AR, amnioreduction; sFGR, selective fetal growth restriction; TAPS, twin anemia‐polycythemia sequence; TRAP, twin reversed arterial perfusion sequence; TTTS, Twin‐twin transfusion syndrome.

During the study period, 616 monochorionic twin pregnancies were evaluated and 265 met the criteria for TTTS. SFLP was performed in 198 subjects, of whom, 17 (8.6%) were excluded from analysis due to the demise of one fetus prior to post SFLP assessment. Further details of the study cohort are depicted in Figure 1.

Of 181 analyzed subjects, mean gestational age at the time of pre‐SFLP assessment was 19.8 (95% CI: ± 0.3) weeks. Pre‐SFLP assessment was performed at a median of 2 (Interquartile range (IQR): 1–4) days prior to intervention. Of these, 9 (5.0%) were Quintero stage I, 105 (58.0%) Quintero stage II, 56 (31.0%) Quintero stage III and 11 (6.1%) Quintero stage IV. Mean CHOP TTTS CV Score for patients identified as Quintero stage I was 5.1 (95% CI: ± 2.1), Quintero stage II was 3.6 (95% CI: ± 0.5), Quintero stage III was 7.0 (95% CI: ± 1.1) and Quintero stage IV was 10.6 (95% CI: ± 3.2).

### 3.1 Overall Cohort

Table 2 shows data for the overall cohort. At the time of pre‐SFLP assessment, donor fetuses demonstrated a higher MCA PI and CPR compared to recipient fetuses (*p* < 0.01 for both). There was no significant difference in UA PI between the fetuses. Post‐SFLP assessment was performed at a median of 7 (IQR: 7–8) days following intervention. Following SFLP, the recipient fetus demonstrated an increase in MCA PI and decrease in UA PI, yielding an increase in CPR. Alternatively, the donor fetus demonstrated a similar magnitude decrease in both the MCA PI and UA PI, yielding no significant change in the CPR PI.

**TABLE 2:** Vascular indices and CHOP TTTS CV score in all subjects with TTTS undergoing SFLP.

	Pre‐SFLP	Post‐SFLP	*p*‐value
All subjects (*n* = 181)			
Vascular indices			
Recipient MCA PI	1.36 (± 0.04)	1.66 (± 0.04)	< 0.01
Recipient UA PI	1.69 (± 0.08)	1.54 (± 0.05)	< 0.01
Recipient CPR	0.87 (± 0.05)	1.12 (± 0.05)	< 0.01
Donor MCA PI	1.84 (± 0.06)	1.74 (± 0.04)	< 0.01
Donor UA PI	1.70 (± 0.07)	1.56 (± 0.05)	< 0.01
Donor CPR	1.15 (± 0.06)	1.16 (± 0.05)	NS
CHOP TTTS CV score elements			
Total CHOP TTTS CV score	5.16 (± 0.56)	3.17 (± 0.47)	< 0.01
Recipient			
Cardiac dilation	0.36 (± 0.08)	0.33 (± 0.08)	NS
Ventricular dysfunction	0.55 (± 0.11)	0.30 (± 0.08)	< 0.01
Ventricular hypertrophy	0.74 (± 0.06)	0.75 (± 0.08)	NS
Tricuspid valve regurgitation	0.66 (± 0.11)	0.44 (± 0.11)	< 0.01
Mitral valve regurgitation	0.20 (± 0.08)	0.09 (± 0.05)	< 0.01
Tricuspid valve inflow	0.40 (± 0.07)	0.19 (± 0.06)	< 0.01
Mitral valve inflow	0.16 (± 0.05)	0.04 (± 0.03)	< 0.01
Ductus venosus flow	0.90 (± 0.12)	0.44 (± 0.10)	< 0.01
Umbilical vein flow	0.27 (± 0.10)	0.08 (± 0.04)	< 0.01
RVOT obstruction	0.26 (± 0.10)	0.18 (± 0.09)	NS
Pulmonary regurgitation	0.31 (± 0.07)	0.14 (± 0.05)	< 0.01
Donor			
Umbilical artery flow	0.42 (± 0.09)	0.22 (± 0.07)	< 0.01

*Note:* All values represent mean (95% confidence interval).

Abbreviations: CHOP TTTS CV Score, Children's Hospital of Philadelphia twin‐twin transfusion cardiovascular score; CPR, cerebroplacental ratio; MCA, middle cerebral artery; PI, pulsatility index; RVOT, right ventricular outflow tract; SFLP, selective fetoscopic laser photocoagulation; UA, umbilical artery.

Following SFLP, the mean total CHOP TTTS CV Score decreased from 5.16 (± 0.56) to 3.17 (± 0.47) (*p* < 0.01). Elements that demonstrated significant early improvement in the recipient twin were systolic dysfunction, tricuspid and mitral regurgitation, tricuspid and mitral inflow pattern, ductus venosus flow pattern, umbilical vein flow pattern and pulmonary regurgitation. Elements that did not improve were cardiac dilation, ventricular hypertrophy, and right ventricular outflow obstruction.

### 3.2 Sub‐Group With Increased CHOP TTTS CVS ≥ 6

The CHOP TTTS CV Score was ≥ 6 in 70 of 198 subjects (35%), of whom 8 were excluded due to a fetal demise. Of the 62 analyzed subjects, the mean gestational age at the time of pre‐SFLP assessment was 20.3 (95% CI: ± 0.3) weeks. Pre‐SFLP assessment was performed at a median of 1 (IQR: 1–2) day prior to intervention. Of these, 4 (7%) were Quintero stage I, 20 (32%) were Quintero stage II, 29 (47%) were Quintero stage III and 9 (15%) were Quintero stage IV. Mean CHOP CV Score for patients identified as Quintero stage I was 7.8 (± 2.0), Quintero stage II was 7.7 (± 0.6), Quintero stage III was 10.2 (± 1.0) and Quintero stage IV was 12.4 (± 2.6).

Table 3 shows data for the sub‐group with CHOP TTTS CV Score ≥ 6. In this subgroup, there were similar but more dramatic changes in vascular indices following SFLP as compared to the overall cohort. CHOP TTTS CV Score elements in this subgroup that demonstrated early improvement were systolic dysfunction, tricuspid and mitral regurgitation, tricuspid and mitral inflow pattern, ductus venosus flow pattern, umbilical vein flow pattern and pulmonary regurgitation. Notably, there was also a statistically significant improvement in cardiac dilation, which was not seen in the overall population. No early improvements were seen in ventricular hypertrophy or right ventricular outflow obstruction.

**TABLE 3:** Vascular indices and CHOP TTTS CV score in subjects with CHOP TTTS CV score ≥ 6 undergoing SFLP.

	Pre‐SFLP	Post‐SFLP	*p*‐value
Sub‐group of CV score ≥ 6 (*n* = 62)			
Vascular indices			
Recipient MCA PI	1.31 (± 0.05)	1.69 (± 0.08)	< 0.01
Recipient UA PI	1.86 (± 0.17)	1.61 (± 0.09)	< 0.01
Recipient CPR	0.77 (± 0.08)	1.09 (± 0.08)	< 0.01
Donor MCA PI	1.89 (± 0.10)	1.74 (± 0.08)	0.04
Donor UA PI	1.74 (± 0.13)	1.52 (± 0.07)	< 0.01
Donor CPR	1.16 (± 0.10)	1.17 (± 0.09)	NS
CHOP TTTS CV score elements			
Total CHOP TTTS CV score	9.55 (± 0.76)	5.16 (± 0.94)	< 0.01
Recipient			
Cardiac dilation	0.87 (± 0.17)	0.65 (± 0.16)	< 0.01
Ventricular dysfunction	1.29 (± 0.18)	0.58 (± 0.18)	< 0.01
Ventricular hypertrophy	0.92 (± 0.07)	0.94 (± 0.06)	NS
Tricuspid valve regurgitation	1.27 (± 0.19)	0.82 (± 0.22)	< 0.01
Mitral valve regurgitation	0.50 (± 0.19)	0.24 (± 0.15)	< 0.01
Tricuspid valve inflow	0.71 (± 0.11)	0.26 (± 0.11)	< 0.01
Mitral valve inflow	0.32 (± 0.12)	0.08 (± 0.07)	< 0.01
Ductus venosus flow	1.42 (± 0.18)	0.63 (± 0.19)	< 0.01
Umbilical vein flow	0.58 (± 0.12)	0.13 (± 0.08)	< 0.01
RVOT obstruction	0.63 (± 0.26)	0.42 (± 0.22)	NS
Pulmonary regurgitation	0.58 (± 0.12)	0.24 (± 0.11)	< 0.01
Donor			
Umbilical artery flow	0.47 (± 0.17)	0.18 (± 0.11)	< 0.01

*Note:* All values represent mean (95% confidence interval).

Abbreviations: CHOP TTTS CV Score, Children's Hospital of Philadelphia twin‐twin transfusion cardiovascular score; CPR, cerebroplacental ratio; MCA, middle cerebral artery; PI, pulsatility index; RVOT, right ventricular outflow tract; SFLP, selective fetoscopic laser photocoagulation; UA, umbilical artery.

## 4 Discussion

This single center cohort study evaluates the early cardiovascular impact of SFLP therapy on both recipient and donor fetuses through measurement of vascular indices and characterization via a detailed cardiovascular scoring system. Doppler measured vascular indices change significantly, reflecting altered cardiovascular loading conditions and there is a significant reduction in fetal cardiovascular burden 1‐week post‐procedure, as assessed by the CHOP TTTS CV Score.

Although treatment with SFLP has led to significantly improved outcomes in pregnancies impacted by TTTS, our understanding of the pathophysiology responsible for the associated cardiac manifestations, particularly in the recipient twin, remains incomplete. Intrinsic to the basis of TTTS is the presence of a vasculopathy within the shared placenta that leads to an inequitable shift of blood and a significant discrepancy in the volume status of each fetus. One twin, the recipient, exists in a hypervolemic state, as compared to that of the hypovolemic donor twin. Intravascular hypovolemia and renal hypoperfusion upregulates the donor twin's renin‐angiotensin system [13], in turn leading to increased peripheral, and potentially, cerebral vascular resistance. While there is increased vascular resistance in donor twins, cardiac structural changes are unusual, unlike the dramatic changes witnessed in the recipient twins. For the recipient, hypervolemia alone cannot account for the cardiac manifestations, as these findings are not consistent with those typically seen in other high cardiac output conditions, such as sacrococcygeal teratomas [14]. While renin production is downregulated in recipient twins, levels of aldosterone are elevated secondary to delivery from the donor fetus through placental anastomoses [15]. The subsequent “dual‐hit” combination of (1) an increased volume preload and (2) a release of hypovolemia‐induced mediators of increased afterload transferred from the donor contributes to the development of a unique recipient fetus cardiomyopathy, manifesting as both systolic and diastolic dysfunction. Blood volume and released vasoactive mediators transmitted from donor to recipient through placental anastomoses lead to the functional and structural change seen in the recipient, while effective interruption of placental anastomoses via SFLP therapy halts the process and allows for cardiovascular recovery.

In our study, vascular Doppler indices confirmed this process. The MCA PI in donor twins, a surrogate for cerebral vascular resistance, is elevated and significantly higher than that in recipient twins. With the redistribution of volume status following SFLP, donor fetuses exhibited a decline of equal magnitude in the PI of both the MCA and UA. These findings are consistent with replenishment of volume and reversal of the pathophysiologic mechanisms of vasoconstriction in response to hypovolemia, as previously described [16]. The recipient twins demonstrated an increase in MCA PI and a decrease in UA PI in response to SFLP therapy. A similar increase in recipient MCA PI following SFLP has been demonstrated on the first post‐procedural day by other investigators [17]. In the recipient, pre‐SFLP MCA PI values may reflect a diminished cardiac output state and hence a compensatory lowering of cerebrovascular resistance to increase cerebral blood flow, along the lines of brain sparing or “cephalization.” With the ameliorative effects of SFLP therapy, it is plausible to suspect that an increase in MCA PI at 1 week is a result of improved cardiac function and hemodynamics.

Use of the CHOP TTTS CV Score to characterize the cardiovascular state confirms an improvement in a host of cardiovascular variables in response to SFLP. Although quantitative indices such as MPI, strain values and others have been used in research studies, such quantitative tools are at risk for measurement error, have demonstrated potential for inter‐observer variability, and often do not correlate with each other [18, 19]. This is why such indices are not readily applied in clinical scenarios in busy imaging laboratories and to date have not been widely incorporated into stratification for clinical decision making in TTTS. No doubt, qualitative measures such as those in the CHOP TTTS CV Score are open to subjective judgments as well; however, the elements included in the score are *all* standard clinical features acquired and assessed during a routine fetal echocardiogram. When interpreted by a dedicated, experienced group of fetal cardiovascular imagers, trained with intent to reduce interpretive variability, it provides an opportunity for a reliable and comprehensive description of the cardiovascular state that can be used for serial surveillance. Of note, our parameter of “single peak” or “double peak” tricuspid valve inflow is a good, readily identified qualitative correlate to the quantitative measure of the myocardial performance index. A single peak, fused inflow pattern reflects a shortened diastolic filling time and a lengthened time interval between tricuspid valve opening and closure, which would result in a higher myocardial performance index reflecting diminished cardiac performance [20]. This is consistent with the findings of other investigators noting improvement in Doppler derived time intervals following SFLP therapy [21].

Several elements of the CHOP TTTS CV Score reflect flow related phenomena. We found that at 1 week following SFLP, all flow related variables significantly improved, except for cardiac dilation. However, even this parameter improved in the higher cardiovascular burden category, suggesting a shift in volume status. Elements which did not change in either the overall cohort or the high cardiovascular burden sub‐group were ventricular hypertrophy and right ventricular outflow obstruction. These are structural variables that would be expected to take some time before remodeling occurs once hemodynamics are altered. Indeed, structural differences may even persist long‐term, in particular abnormalities such as pulmonary stenosis or atresia [22, 23]; thus, it is not surprising that we did not see a significant change in this element in our study given the focus on the early post‐SFLP assessment. Of note, we have incorporated a unique finding of relative main pulmonary artery size to aortic size as an important element to describe in the recipient fetus. In the normal heart, the main pulmonary artery is always larger than the aorta; however, we have found that the recipient fetus in TTTS can manifest changes in pulmonary artery size with diminution relative to the aorta. This is part of an early change in cardiac structure related to the dynamic flow alterations taking place in that fetus. Main pulmonary artery diminution is an early marker of right ventricular outflow change and is a manifestation at one end of the spectrum of right‐sided structural change, with structural pulmonary atresia at the other. We have seen a change in main pulmonary artery size, as well as states of “functional” pulmonary atresia resolved dramatically in response to SFLP therapy. Both represent findings responsive to acute shifts in loading conditions. However, when present, structural pulmonary atresia is a fixed development that will not change with SFLP and must be treated after birth [24].

Interestingly, we found a slightly higher CHOP TTTS CV Score in our Quintero stage I than in our stage II subjects (5.1 vs. 3.6). Indeed, there is bias, as our practice selectively offers SFLP to those with Quintero stage I only if there are significant cardiovascular findings or increased risk for pregnancy loss. However, this precisely demonstrates the point that the Quintero staging system does not incorporate important cardiovascular abnormalities. These features are actively present and influence the disease pathophysiology, far in advance of reaching Quintero stage III, defined as manifesting Doppler abnormalities. There are no cardiovascular variables included in Quintero stage I or II. A careful cardiovascular focused evaluation such as that offered by the CHOP TTTS CV Score reveals numerous findings early in the conventional Quintero staging of TTTS disease [25] and, in our view, should be incorporated in the initial and serial assessment of the condition as well as therapeutic management decision‐making [26].

We acknowledge several limitations to our study. To permit paired analysis of donor and recipient twins, we excluded subjects with demise following SFLP of one or both fetuses. In doing so for this study, we were not able to evaluate pre‐ or post‐SFLP features that may provide insight into those pregnancies at greater risk for the demise of one or both fetuses. Second, a small subset of fetuses developed twin anemia‐polycythemia sequence (TAPS) following SFLP (6%). In our experience, when TAPS and recurrent TTTS develop, this occurs after the first week of follow‐up. Therefore, we do not believe this would have greatly impacted evaluation at 1 week following SFLP, though acknowledge the potential effect in a very small subset of fetal dyads.

All post‐SFLP data were collected 1 week post‐procedurally; hence, we cannot comment on the precise timeline of improvement, which may potentially occur as early as one day after the laser. Serial post‐SFLP assessments would provide additional insight into the progressive changes to both vascular indices and individual elements of the CHOP TTTS CV Score.

Finally, this is a single center study utilizing our own developed scoring system with subjective measures included. Generalizability to other centers may be limited. Nevertheless, while the tool may be limited in this respect, we have demonstrated that through our application, it points to the development of dramatic physiological changes in the acute post‐laser period that invites further curiosity concerning the biology of this disease.

## 5 Conclusion

In summary, there is significant improvement in cardiovascular burden as early as 1 week following SFLP therapy. Acute changes in flow related cardiovascular variables in our scoring system, the CHOP TTTS CV Score, further confirms the origins and mechanisms of the disease as related to abnormal loading conditions that are acutely modified through SFLP therapy. The CHOP TTTS CV Score, through detailed cardiovascular characterization, is a tool that complements traditional Quintero staging. Enhanced characterization of the cardiovascular turmoil of TTTS may allow for better study of the potential impact of this condition through fetal programming on long‐term outcomes and lifelong health [27].

## Conflicts of Interest

The authors declare no conflicts of interest.

## Data Availability

The data that support the findings of this study are available from the corresponding author upon reasonable request.
